# Radiation risk factors in incidence and mortality among exposed individuals of East Kazakhstan

**DOI:** 10.5195/cajgh.2013.105

**Published:** 2014-01-24

**Authors:** Kazbek Apsalikov, Talgat Muldagaliev, Rustem Apsalikov, Shinar Serikkankyzy, Zaure Zholambaeva

**Affiliations:** Scientific Research Institute for Radiation Medicine and Ecology, Kurchatov, Kazakhstan

**Keywords:** ionizing radiation, radiation risk, Kazakhstan

## Abstract

**Introduction:**

Lengthy clinical and epidemiological studies at the Research Institute of Radiation Medicine and Ecology have discovered basic patterns of long-term effects from ionizing radiation in population groups exposed to radiation risk. Methodology for calculating injury from radiation risk factors has been developed and implemented to minimize the effects of the Semipalatinsk nuclear test site (SNTS).

**Material and methods:**

We analyzed materials from the database of the Scientific Medical Register that were exposed to radiation as a result of SNTS. We analyzed both male and female populations of the Abay, Beskaragai and Zhanasemei, Kokpekti (control) areas of East-Kazakhstan region (EKR) from 2008–2012. These populations were split into three groups allocated by the generation. The first group represented persons born from the period of 01/01/1930–08/01/1949 and their children born from the period of 10/09/1949–12/31/1962. The second group were persons born after 01/01/1963. The third group served as the control and were persons who immigrated to these areas after 1990.

**Results:**

There was an increased incidence of cancer (21.5%, p < 0.000734), cardiovascular diseases (10.2%); respiratory problems (9.6%), gastrointestinal issues (9.1%, p < 0.00371–0.00679) in the first group. The effect of the radiation dose has not been fully stuided among the subjects in the second group.

The major causes of excess mortality in the first group were neoplams (30.6%), hypertension (23.8%), and myocardial infarction (22.6%). The effects of radiation influenced mortality in the second group were 2–2.5 times lower than the first group.

**Conclusion:**

There is a correlation between the size of the radiation dose, the risk profile, and age at the moment of radiation exposure with trends of morbidity and mortality in the radiation exposed areas.

